# The application of Chicago classification 4.0 in esophageal motility disorders of scleroderma: a case-control study in China

**DOI:** 10.3389/fimmu.2026.1962332

**Published:** 2026-10-05

**Authors:** Yinghui Yang, Siwei Xie, Minyi Xu, Zhiming Lin, Yanli Zhang

**Affiliations:** 1 Department of Gastroenterology, The Third Affiliated Hospital of Sun Yat-sen University, Guangzhou, China; 2 Johns Hopkins University School of Medicine, Baltimore, MD, United States; 3 Department of Rheumatology and Immunology, The Third Affiliated Hospital of Sun Yat-sen University, Guangzhou, China

**Keywords:** Chicago v4.0, high-resolution manometry, imaging of esophageal motility disorders, lung dysfunction, systemic sclerosis

## Abstract

**Background:**

Systemic sclerosis (SSc) is a rare autoimmune connective tissue disease characterized by multi-organ fibrosis and vasculopathy that frequently involves the esophagus. This study assessed esophageal motility disorders in patients with SSc using High-resolution manometry (HRM) and classified these disorders according to the Chicago Classification version 4.0, aims to investigate the associations between esophageal dysfunction and clinical phenotypes in Chinese SSc patients, while exploring potential modifications to enhance the applicability of the Chicago v4.0 protocol in SSc patients.

**Methods:**

We retrospectively identified SSc patients fulfilling 2013 ACR criteria who underwent HRM classified by Chicago v4.0 at our center (2021–2023). Demographics, organ involvement, pulmonary-function tests, echocardiography and reflux symptom scores (RDQ/GERDQ) were extracted from the hospital information system; age and sex matched non-SSc HRM subjects served as controls. Associations between Chicago categories and clinical variables were examined by multinomial logistic regression.

**Results:**

In our study, esophageal motility disorders were present in 76.7% of patients with systemic sclerosis, predominantly characterized by ineffective esophageal motility (IEM) (35%) and absent contractility (AC) (36.67%). The occurrence rate of AC rose with age. Symptoms of gastroesophageal reflux were more common in the AC group [Odds Ratio: 3.23 (1.02-10.22) vs 1.43(0.52-3.95), P<0.05, E value=2.99]. Diffusion capacity for carbon monoxide (DLCO) was significantly reduced in both IEM and AC (-2.03 vs -2.70, P value<0.05), with a more pronounced decrease in AC. Total lung capacity (TLC) and forced expiratory volume in one second (FEV1) exhibited stronger associations with AC (-1.04 and -0.8 respectively, p value<0.05). Upright HRM revealed more marked decreases in EGJ pressure and distal contractile integral (DCI) relative to the supine position in SSc patients, indicating that esophageal abnormalities may be more readily apparent during upright examination.

**Conclusions:**

Esophageal motility disorders in SSc were common and significantly associated with older age, gastroesophageal reflux symptoms, and reduced lung function. Future studies are needed to clarify whether early esophageal motility disorders are associated with disease progression and prognosis in patients.

## Highlights

- This is the first study to apply Chicago Classification 4.0 in scleroderma patients in China.
- The findings provide new insights into esophageal motility patterns specific to this population.
- These results may contribute to our understanding of clinical management and guide future research in this field.

## Introduction

Systemic sclerosis (SSc) is a rare autoimmune connective tissue disease characterized by multi-organ fibrosis and vasculopathy. Variation in its incidence and prevalence seems to be race and geography specific (1–7), Large population- and registry-based cohorts from Europe and North America have further characterized the epidemiology, clinical phenotypes, and gastrointestinal involvement of SSc in different populations (8–12). But nationwide epidemiological data remains absent in China. Prevalence in Asia ranges from approximately 30 to 90 cases per million individuals, with annual incidence of 7 to 15 cases per million person-years. These figures vary considerably depending on the diagnostic and classification criteria employed. It is important to note that prevalence and incidence estimates vary substantially according to the classification criteria used (e.g., 1980 ARA criteria vs. 2013 ACR/EULAR criteria), making direct comparisons between studies challenging. (13, 14).

The gastrointestinal tract is often implicated in SSc, with the esophagus being the organ most frequently affected. Multi-ethnic studies have suggested racial disparities in esophageal manifestations, with Asians exhibiting lower rates of esophageal dysmotility than Caucasians (13, 15). Erosive esophagitis was observed in patients with higher frequency, especially in those with absent contractility (16). Some studies have reported that esophageal dysfunction may be associated with pulmonary involvement in scleroderma (17). The mechanism is thought to be related to poor esophageal motility and reduced lower esophageal sphincter (LES) pressure, which can lead to gastroesophageal reflux and micro-aspiration into the airways (18–20). However, research on these mechanisms in the Chinese population remains limited. Recent studies have further characterized esophageal motor dysfunction in SSc, identifying distinct dysmotility patterns associated with clinical phenotypes and prognosis, and proposing risk stratification models based on manometric findings (12, 21, 22), as well as objective gastrointestinal involvement in large SSc registries (21–23).

High-resolution manometry (HRM) has emerged as a milestone in the field of esophageal motility evaluation, offering a more accurate and comprehensive diagnostic tool compared to traditional methods. During manometric assessments, the classic sclerotic esophagus typically exhibits an absence of peristalsis in the lower esophageal segments and reduced pressure in the LES. The Chicago Classification version 4.0, published in 2020, provides a standardized algorithmic framework for the categorization of esophageal motility disorders based on HRM metrics (24).

Despite these advancements, the ethnic-specific patterns of esophageal involvement in Chinese SSc patients remain poorly characterized within the Chicago Classification 4.0 framework. This research aims to investigate the associations between esophageal dysfunction and clinical phenotypes in Chinese SSc patients, while exploring potential modifications to enhance the applicability of the Chicago 4.0 protocol in this specific patient population.

## Methods

### Patients and ethnics

A single-centre retrospective analysis was performed on 70 SSc cases that fulfilled the 2013 American College of Rheumatology classification criteria for SSc (25) and underwent HRM examination in accordance with the Chicago v4.0 from January 2021 to October 2023 in the Third Affiliated Hospital of Sun Yat-sen University. Ten cases were excluded from the analysis due to incomplete HRM examinations resulting from patients intolerance or lack of cooperation. Clinical and demographic profiles, including age, sex, age onset, duration of disease, SSc-related clinical features such as Raynaud’s phenomenon, Modified Rodnan skin score (mRSS), blood tests, pumonary CT scan evaluation, pulmonary function tests, heart ultrasound and esophageal symptoms were sourced from the hospital’s information system. Reflux Disease Questionnaire (RDQ) and Gastroesophageal Reflux Disease Questionnaire (GERDQ) were employed to gather data on upper gastrointestinal symptoms among patients with scleroderma. The control group comprised non-scleroderma outpatients who were clinically referred for high-resolution manometry (HRM) because of suspected gastroesophageal reflux disease (GERD), achalasia, or other esophageal motility disorders. Common referral symptoms included acid regurgitation, heartburn, belching, retrosternal discomfort, chest pain, dysphagia, cough, and throat discomfort. The study was approved by the Medical Ethics Committee of the Third Affiliated Hospital of Sun Yat-sen University (SL-II2023-123-02) and informed consents were obtained from all participants. HRM was performed as part of a standardized local practice for SSc patients with suspected esophageal involvement, including those presenting with reflux symptoms, dysphagia, or as part of routine screening for gastrointestinal involvement in newly diagnosed SSc patients. Data on SSc subtypes (diffuse cutaneous SSc [dcSSc] and limited cutaneous SSc [lcSSc]), Raynaud’s phenomenon, digital ulcers, and current anti-reflux therapies (proton pump inhibitors, histamine-2 receptor antagonists) were systematically collected. While due to the small sample size, no statistically significant differences of subtypes and therapies were observed in the statistical analysis.

### High-resolution manometry procedure

Esophageal high-resolution manometry was conducted using a solid-state catheter with 36 sensors at 1-cm intervals. The following manometric parameters were assessed for classification per the Chicago Classification v4.0: basal lower esophageal sphincter pressure, integrated relaxation pressure (IRP), distal contractile integral (DCI), and distal latency (DL), Multiple Rapid Swallow (MRS).

### Statistical analysis

For descriptive comparisons, we used the Shapiro-Wilk test to check whether continuous variables followed a normal distribution. When the data met normality assumptions, independent t-tests were used to compare groups. If the distribution was not normal, the Wilcoxon rank-sum test was chosen instead. For categorical variables, Fisher’s exact test was applied in cases with small sample sizes (usually fewer than 5 observations per categorical type), while chi-square tests were used when the sample size was larger.

Variables included in the regression analyses were selected *a priori* based on their clinical relevance to systemic sclerosis and esophageal involvement, previous literature, and data availability. Given the exploratory objective of the study and the available sample size, each clinical characteristic was evaluated separately. Separate univariable multinomial logistic regression models were used to evaluate the associations between individual clinical characteristics and the Chicago Classification categories, with odds ratios (ORs) and corresponding 95% confidence intervals (CIs) reported. Separate univariable linear regression models were used to evaluate the associations between Chicago Classification categories and continuous clinical outcomes, including pulmonary-function parameters, pulmonary artery systolic pressure (PASP), and the modified Rodnan skin score (mRSS). For selected regression analyses, we additionally calculated the E-value for each association to evaluate the potential influence of unmeasured confounding.

All data cleaning, preprocessing, and statistical analyses were performed using the R software (version 4.4.2, R Project for Statistical Computing). A two-tailed p-value threshold of less than 0.05 was applied to determine statistical significance. Given the exploratory nature of this study and the limited sample size, we did not apply formal correction for multiple comparisons (e.g., Bonferroni or false discovery rate). Therefore, the results should be interpreted as hypothesis-generating, and p-values close to 0.05 should be interpreted with caution. We have added E-values for selected associations to provide additional context for the robustness of our findings against unmeasured confounding.

## Results

### Demographic and clinical characteristics

Patients with SSc enrolled were mostly female (75%), with a median age of 49.5 years (interquartile range [IQR]: 42.5 -56.25 years). Their nutritional status was within the normal range, with a median body mass index (BMI) of 21.35 (IQR: 18.88 -23.04). The median age of onset was 46 years (IQR: 33.00 -52.00 years), and the median disease duration from first SSc symptom onset was 4 years (IQR: 2–7 years). The median mRSS score was 10 (IQR: 6-17.5). Pulmonary CT scan reported 12 (20%) patients with interstitial pneumonia. PASP were detected to be 31.00 (IQR: 26.00-36.00) (**Table 1**). Among the study cohort, 14 patients (23.3%) reported no upper gastrointestinal symptoms. The predominant clinical manifestations were reflux-related, with acid regurgitation or heartburn reported in 40% of cases and dysphagia in 26.67% (**Table S1**). Despite reporting reflux symptoms, the patients’ self-assessed RDQ scores (IQR: 2.5; threshold≥12 suggested gastroesophageal reflux disease) and GERDQ scores (IQR: 1.0; threshold≥8 indicated a possible diagnosis of gastroesophageal reflux disease) were both below the diagnostic thresholds (**Table 1**).

**Table 1:** Demographic and clinical characteristics of SSc patients.

Characteristic	SSc (N = 60)	Control (N = 60)	P value
Female, n (%)	45 (75%)	36 (60%)	0.118
Age, years, median (IQR)	49.50 (42.50-56.25)	46.00 (31.75-56.75)	0.241
BMI, Median (IQR)	21.35 (18.88-23.04)	22.39 (19.95-24.22)	0.068
Duration of disease, years, median (IQR)	4.00 (2.00-7.00)	-–-	
Onset Age, years,	46.00 (33.00-52.00)	-–-	
mRSS, median (IQR)	10.00 (6.00-17.50)	-–-	
DLCO, median (IQR)	5.11 (3.96-5.85)	-–-	
TLC, median (IQR)	3.80 (3.28-4.37)	-–-	
RV, median (IQR)	1.61 (1.43-1.82)	-–-	
FVC, median (IQR)	2.41 (1.80-2.70)	-–-	
FEV1, median (IQR)	1.93 (1.56-2.48)	-–-	
Interstitial Pneumonia, n (%)	12 (20.00)	-–-	
PASP, median (IQR)	31.00 (26.00-36.00)	-–-	
Pericardial Effusion	6 (10.00)	-–-	
LVEF %, median (IQR)	68.00 (64.00-72.00)	-–-	
RDQ score, median (IQR)	2.50 (0.00-7.25)		
GERDQ score, median (IQR)	1.00 (0.00-4.25)		
ANA positive, n (%)	54 (90%)	-–-	
Scl-70 positive, n (%)	25(41.67)	-–-	
Anti-Centromere Antibody, n (%)	8 (13.33)	-–-	

BMI, Body mass index; IQR, interquartile range; mRSS, Modified Rodnan skin score; DLCO, Diffusion capacity for carbon monoxide; TLC, Total lung capacity; RV, Residual volume; FVC, Forced vital capacity; FEV1, Forced expiratory volume in one second; PASP, Pulmonary artery systolic pressure; LVEF, Left ventricular ejection fraction;ANA, Antinuclear antibodies.

The laboratory tests including complete blood count, liver function, renal function, blood lipids, muscle enzymes, Inflammatory markers (C3, C4, CH50, IgG, CRP, ESR) were all within the normal range (**Supplementary Table S2**). An additional table shows this in more detail [**Supplementary file**]. Antinuclear antibodies (ANAs) were the most common type of autoantibody detected (90%). However, no correlation was found between the titer of ANAs, other autoantibodies, and the classification of esophageal motility disorders.

### HRM characteristics of SSc patients

According to the Chicago Classification 4.0 criteria for high-resolution manometry (HRM), significant differences were observed between SSc patients and the symptomatic HRM controls (**Table 2**). The prevalence of ineffective esophageal motility was markedly higher in SSc patients (35%) compared to controls (15%), while absent contractility was also significantly more frequent in SSc patients (36.67%) than in controls (5%) (**Table 2**). Typical esophageal aperistalsis in systemic sclerosis (SSc) is schematically illustrated in **Figures 1A–D**. An additional movie file shows this in more detail [**Additional file 1**, **Additional file 2**]. Furthermore, the analysis identified two cases of Type I achalasia and one case of esophagogastric junction outflow obstruction among the SSc cohort. The LES Length in intra-abdominal (upright position) region was shorter in SSc patients compared to controls. The Esophagogastric junction (EGJ)pressure [2.90 (0.15-12.65) vs 9.00 (4.35-18.13) expiration respectively, P = 0.002; 10.10 (4.28-17.58) vs 15.45 (6.90-25.03) inspiration respectively, P = 0.014] and EGJ-CI in the upright position were significantly lower than those of the symptomatic HRM controls [1.6 (0.10-32.10) vs 24.85 (0.90-117.15) respectively, P = 0.007], whereas these differences were not statistically significant in the supine position. Median Integrated relaxation pressure (IRP), Distal contractile integral (DCI) were significantly lower than control values in both positions, irrespective of the bolus type (water or colloid) administrated (**Table 2**). The disappearance of striated muscle contraction was less frequent in SSc patients [57 (95.00%) vs 46 (76.67%) respectively, P = 0.007].

**Table 2:** Comparison of HRM characteristics of SSc patients and controls.

HRM characteristic	Scleroderma (N = 60)	Control (N = 60)	P value
Ineffective esophageal motility, n (%)	21 (35.00)	9 (15.00)	
Absent contractility, n (%)	22 (36.67)	3 (5.00)	
Achalasia, n (%)	2 (3.33)	11 (18.33)	
EGJ Outflow Obstruction, n (%)	1 (1.67)	2 (3.33)	
Esophageal pressure test data, median (IQR)			
LES Length (Supine Position), median (IQR)	3.80 (2.90-4.70)	3.95 (3.00-4.73)	0.570
LES Length in Intra-abdominal (Supine Position)	2.80 (2.30-3.80)	3.00 (2.48-3.73)	0.551
LES Length (Upright Position), median (IQR)	3.70 (2.50-4.75)	4.00 (3.00-5.03)	0.124
LES Length in Intra-abdominal (Upright Position)	2.50 (1.90-3.50)	3.10 (2.50-4.23)	0.002
EGJ Pressure end of expiration (Supine Position)	9.35 (4.93-15.83)	12.65 (6.65-19.13)	0.045
EGJ Pressure end of expiration (Upright Position)	2.90 (0.15-12.65)	9.00 (4.35-18.13)	0.002
EGJ Pressure end of inspiration (Supine Position)	18.20 (9.68-26.45)	20.75 (16.20-28.38)	0.061
EGJ Pressure end of inspiration (Upright Position)	10.10 (4.28-17.58)	15.45 (6.90-25.03)	0.014
EGJ-CI (Supine Position), median (IQR)	42.75 (6.95-114.88)	63.40 (20.90-208.65)	0.071
EGJ-CI (Upright Position), median (IQR)	1.60 (0.10-32.10)	24.85 (0.90-117.15)	0.007
UES Pressure (Supine Position), median (IQR)	56.10 (42.75-75.70)	73.25 (53.33-109.70)	0.001
UES Pressure (Upright Position), median (IQR)	52.00 (32.80-76.53)	49.60 (36.15-62.00)	0.388
UES Relaxation Pressure (Supine Position)	5.05 (0.53-10.40)	5.50 (1.18-12.13)	0.175
UES Relaxation Pressure (Upright Position)	1.80 (-3.43-5.25)	-1.55 (-3.78-2.08)	0.091
IRP Median (Supine Water Test), median (IQR)	6.95 (3.71-11.23)	9.33 (5.03-16.30)	0.034
IRP Median (Supine Colloid Test), median (IQR)	5.75 (3.25-12.25)	8.75 (5.68-17.55)	0.010
IRP Median (Upright Water Test), median (IQR)	2.75 (0.50-4.90)	3.40 (1.20-9.23)	0.080
IRP Median (Upright Colloid Test), median (IQR)	2.00 (0.10-3.98)	3.90 (1.38-10.60)	0.003
DCI (Supine Water Test), median (IQR)	85.07 (10.57-378.26)	830.52 (463.32-1506.90)	<0.001
DCI (Supine Colloid Test), median (IQR)	59.80 (2.90-223.03)	750.65 (374.16-1316.14)	<0.001
DCI (Upright Water Test), median (IQR)	9.53 (0.02-113.44)	439.92 (199.05-910.19)	<0.001
DCI (Upright Colloid Test), median (IQR)	2.89 (0.10-87.75)	519.75 (176.33-873.93)	<0.001
Striated Muscle Contraction, n (%)	57 (95.00)	46 (76.67)	0.007
Proximal Smooth Muscle Contraction, n (%)	33 (55.00)	42 (70.00)	0.131
Mid-Distal Smooth Muscle Contraction, n (%)	27 (45.00)	43 (71.67)	0.005
LES Relaxation during Swallowing, n (%)	50 (83.33)	45 (75.00)	0.369
MRS Contraction, n (%)	20 (33.33)	44 (73.33)	<0.001
MRS-DCI, median (IQR)	29.85 (2.58-242.03)	1400.80 (655.98-2635.58)	<0.001
MRS-IRP, median (IQR)	5.70 (2.58-8.40)	6.90 (3.70-12.78)	0.032

LES, Lower esophageal sphincter; EGJ, Esophagogastric junction; UES, Upper Esophageal Sphincter; DCI, Distal contractile integral; IRP, Integrated relaxation pressure; MRS, Multiple rapid swallows. The control group comprised non-scleroderma outpatients with suspected GERD/esophageal motility disorders undergoing HRM.

**Figure 1:**
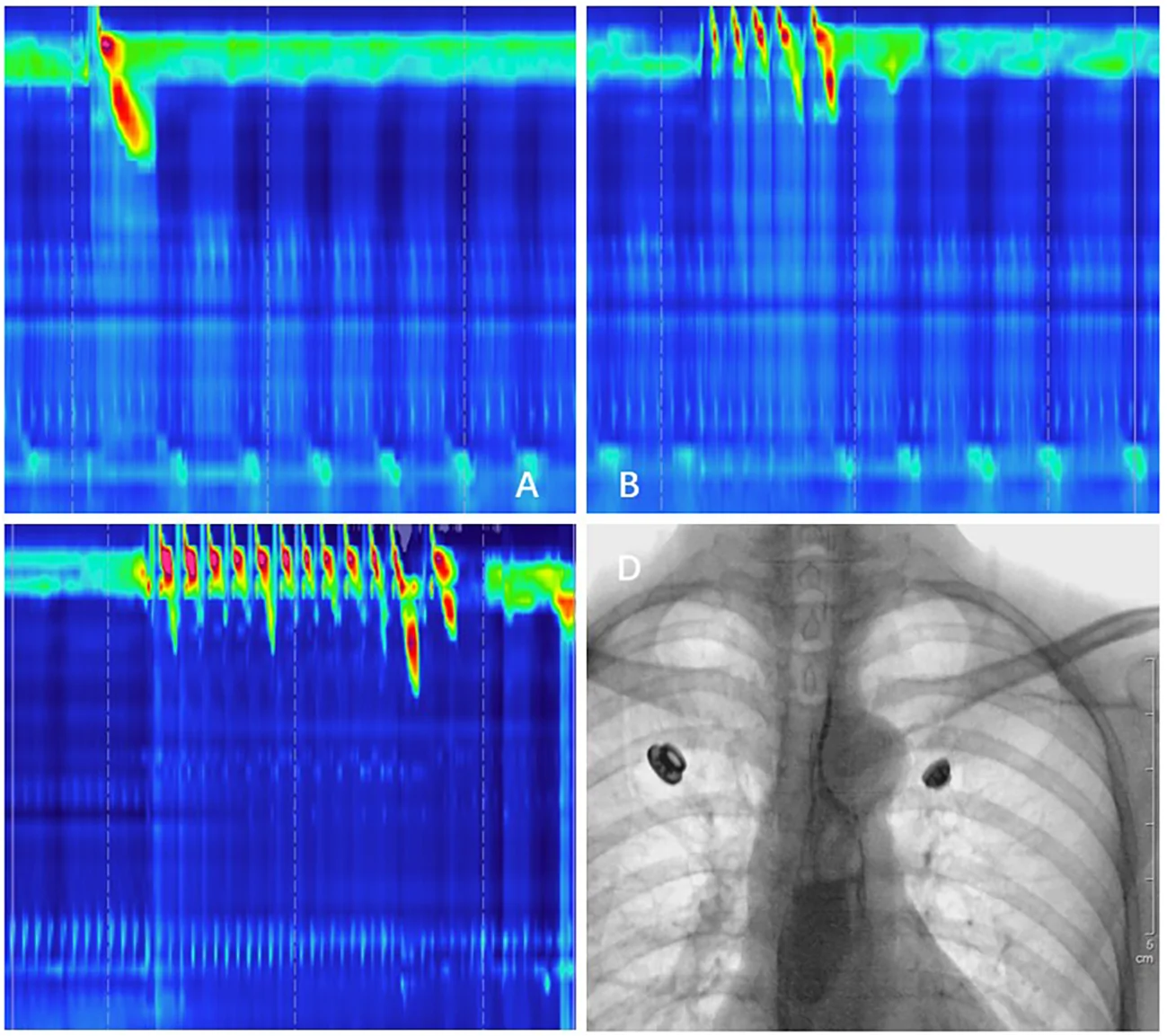
HRM findings in patients with SSc. **(A)** Single water swallow reveals absent esophageal smooth muscle motility. **(B)** No esophageal peristalsis was elicited by MRS. **(C)** RDC showed a decreased IRP. **(D)** Upper gastrointestinal series revealed complete absence of peristalsis in the middle and lower esophagus.

Similarly, the absence of contraction during MRS was more frequent in SSc patients. Both Multiple rapid swallows distal contractile integral (MRS-DCI) and Multiple rapid swallows Median Integrated relaxation pressure (MRS-IRP) were significantly reduced compared with the symptomatic HRM controls (**Table 2**).

### The relationship between HRM Chicago classification and clinical manifestations

We divided SSc patients into five groups based on their esophageal manometry results: 14 in the normal group(23.3%), 21 in the IEM group(35%), 22 in the AC group(36.7%), 2 in the achalasia group(3.3%), and 1 in the outflow obstruction group(1.7%). Owing to the inadequate sample sizes for Type I achalasia and esophagogastric junction outflow obstruction, these subtypes were omitted from the multinomial logistic regression analysis. Gender and BMI were not associated with IEM or AC. The incidence of AC increased with age and age at onset. Among upper gastrointestinal symptoms, acid regurgitation, heartburn, and dysphagia were more common in the AC group. [Odds Ratio: 3.23 (1.02-10.22) vs 1.43(0.52-3.95), P<0.05, E value=2.99], while symptoms such as globus sensation, upper abdominal pain, bloating, belching, and nausea were not related to the classification type. The RDQ score was higher in the AC group [Odds Ratio: 3.68 (1.44-9.39) vs 2.12 (0.93-4.84), P<0.05, E value=3.24], while there was no significant difference in the GERD score between the groups (**Figure 2**). Therefore, it is difficult to assess the severity of esophageal involvement and esophageal motility impairment based on patients’ self-reported reflux symptoms. However, symptoms such as acid regurgitation, heartburn, and dysphagia may be helpful for clinicians to identify more severe esophageal involvement. DLCO was significantly reduced in both IEM and AC (-2.03 vs -2.70, P value<0.05), with a more pronounced decrease in AC (**Table 3**). TLC and FEV1 exhibited stronger associations with AC (-1.04 and -0.8 respectively, p value<0.05). No statistical differences were detected in pulmonary arterial hypertension, pulmonary interstitial fibrosis, or mRSS (**Table 3**).

**Figure 2:**
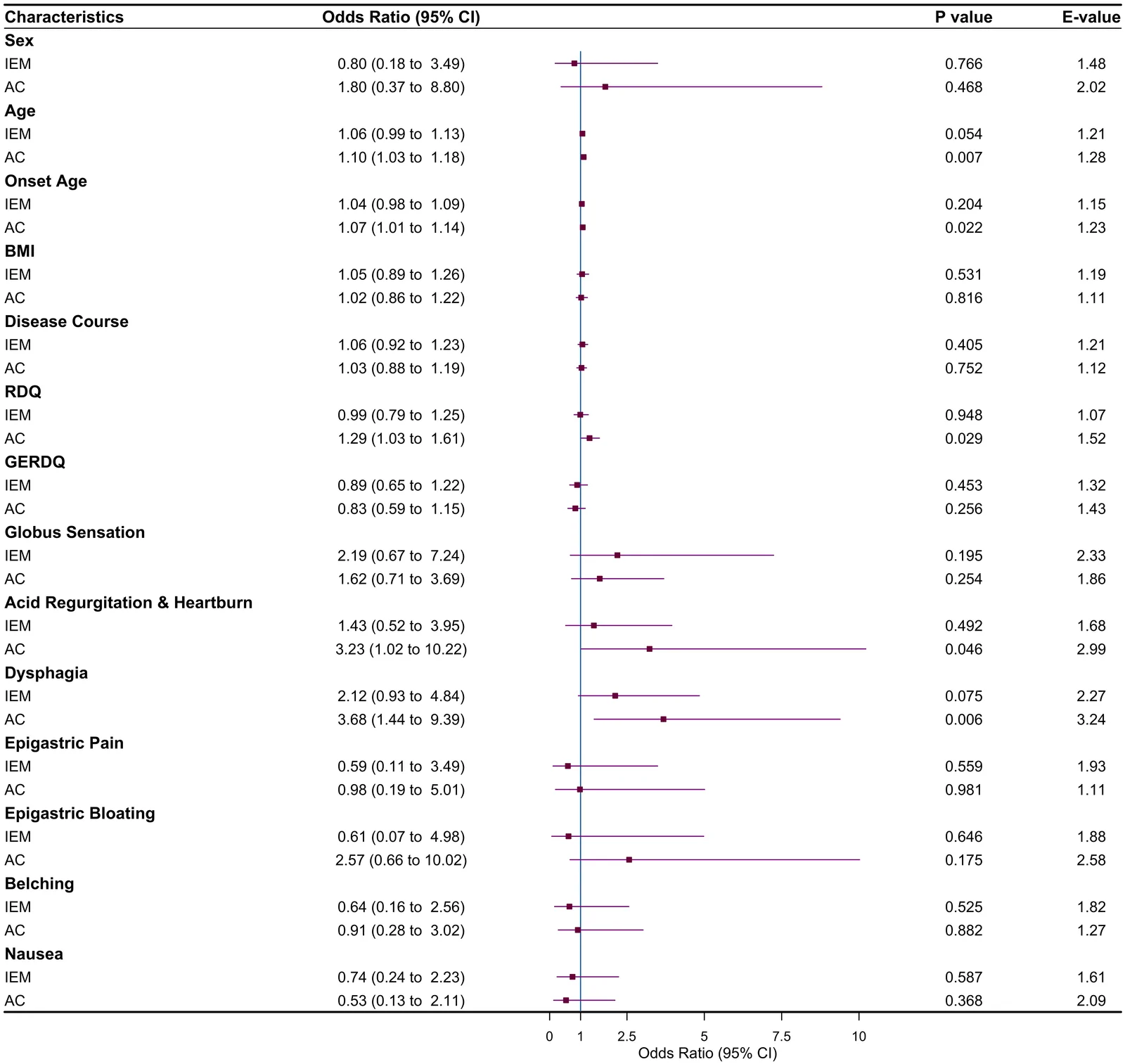
Multinomial logistic regression analyses of the associations between individual clinical characteristics and Chicago Classification version 4.0 categories.

**Table 3:** Association between Chicago classification categories and clinical measurement outcomes.

Clinical measurement outcome/Chicago Classification v4.0 category	Coefficient	SE	95% CI	P value
DLCO				
IEM	-2.03	0.73	-3.46 to -0.60	0.009
AC	-2.70	0.71	-4.09 to -1.31	0.001
TLC				
IEM	-0.58	0.41	-1.39 to 0.22	0.166
AC	-1.04	0.40	-1.82 to -0.25	0.014
RV				
IEM	-0.35	0.18	-0.70 to 0.01	0.060
AC	-0.36	0.17	-0.70 to -0.01	0.050
FVC				
IEM	-0.48	0.67	-1.79 to 0.83	0.474
AC	-0.52	0.66	-1.81 to 0.76	0.430
FEV1				
IEM	-0.48	0.35	-1.16 to 0.19	0.170
AC	-0.80	0.34	-1.46 to -0.14	0.024
PASP				
IEM	-1.02	3.15	-7.20 to 5.16	0.748
AC	4.23	3.15	-1.95 to 10.41	0.187
mRSS				
IEM	-1.15	2.63	-6.31 to 4.00	0.663
AC	2.85	2.61	-2.26 to 7.95	0.280

IEM, Ineffective esophageal motility; AC, Abolition of contraction; mRSS, Modified Rodnan skin score; DLCO, Diffusion capacity for carbon monoxide; TLC, Total lung capacity; RV, Residual volume; FVC, Forced vital capacity; FEV1, Forced expiratory volume in one second; PASP, Pulmonary artery systolic pressure.

## Discussion

A study on ethnic variations in systemic sclerosis disease manifestations showed that, compared with European-descent whites, East Asians had a lower prevalence of esophageal dysmotility (88% vs 69%) (15). In a study from South Korea, the proportion was 60.9% (16). In our study, esophageal dysmotility (including absent contractility, ineffective esophageal motility,Type I achalasia, and outflow obstruction) accounted for 76.7% (46/60).

SSc is prone to misdiagnosis in its early stages. The relatively short disease duration in the SSc population of this study suggests that esophageal motility disorders in SSc patients may manifest as symptoms of gastroesophageal reflux and dysphagia in the early stages, or may be asymptomatic. Therefore, clinicians should be vigilant for the possibility of SSc in patients presenting with initial complaints of gastroesophageal reflux or dysphagia, particularly if HRM reveals absent esophageal smooth muscle motility, with or without reduced LES pressure at rest. It is recommended to conduct a detailed medical history and physical examination to rule out SSc. Additionally, the potential for esophageal involvement should not be overlooked in SSc patients who are asymptomatic in the upper gastrointestinal tract.

Based on the Chicago Classification version 4.0, upright HRM demonstrated more pronounced reductions in EGJ pressure and esophageal contractility than the supine examination. Because the diagnoses of absent contractility and esophagogastric junction outflow obstruction require combined interpretation of both supine and upright examinations according to the Chicago Classification version 4.0, these findings should be interpreted as demonstrating more apparent physiological abnormalities during upright examination rather than higher diagnostic sensitivity. Meanwhile, using colloid swallows or not appears to have little impact on the results of DCI, MRS, and IRP. Therefore, for SSc patients with poor tolerance, a water swallow test alone may be attempted to shorten the examination time and reduce patient discomfort.

Cardiopulmonary diseases are the most significant threat to patients with SSc, representing a high-risk factor that severely impacts the prognosis of SSc and emerging as the leading cause of death in these patients. Previous studies have shown that the severity of esophageal motility disorders, detected by conventional manometry or HRM, correlates with evidence of ILD on pulmonary function tests and HRCT scans (17, 26, 27). Longitudinal studies with repeated esophageal HRM and HRCT scans have revealed that lower LES pressure is associated with more severe pulmonary involvement on follow-up CT scans years later, leading to the speculation that gastroesophageal reflux may be a precipitating factor for interstitial lung disease in SSc (28). Consistent with these findings, our study also demonstrated that esophageal motility disorders, particularly AC, are significantly associated with decreased lung function, such as reduced DLCO, TLC and FEV1. These results support previous research suggesting that esophageal motility disorders may lead to gastroesophageal reflux and micro-aspiration, thereby triggering or exacerbating pulmonary fibrosis. However, our retrospective cross-sectional design precluded testing whether esophageal motility disorders cause lung injury via reflux or micro-aspiration, given the inconsistent availability of endoscopy, pH monitoring, and longitudinal pulmonary imaging. A study in South Korea observed erosive esophagitis in 12.2% of SSc patients and found that AC was more common in patients with erosive esophagitis than in those without (80.0% vs 13.9%). Another research observed AC was associated with regurgitations and erosive esophagitis (29). However, due to the limited use of endoscopy and 24-hour pH monitoring in our study, we were unable to integrate endoscopic findings, esophageal pH monitoring and manometry to determine whether gastroesophageal reflux in SSc patients is associated with lung function and pulmonary fibrosis. The newer wireless esophageal pH monitoring, with its better tolerance, may become a future option for us. The Functional Lumen Imaging Probe (FLIP) is another tool employing impedance planimetry to evaluate the dimensions and distensibility of the esophagus under controlled volumetric distension. Carlson et al. combined FLIP Panometry and HRM assessment in a cohort of SSc patients, demonstrating heterogeneity in primary and secondary peristalsis, try to proposed a new classification model for “scleroderma esophagus” (30). Novel methods of esophageal pH monitoring and functional assessment may offer more precise diagnostics for the esophageal involvement in SSc.

Johns Hopkins Scleroderma Center (JHSC) Research found AC was observed in most of the SSc patients who died, but these findings were not seen in patients with IEM. Our study is retrospective, with a relatively short disease duration among the included patients and fewer occurrences of severe complications or comorbidities. Prospective studies are needed to observe whether the classification of motility disorders can predict complications and prognosis, including their relationships with pulmonary interstitial fibrosis, pulmonary arterial hypertension, cardiovascular involvement, and mortality. Additionally, interventional treatments such as immunosuppressive agents and plasma exchange should be investigated for their potential impact on reflux symptoms and esophageal motility. It is also important to explore whether anti-reflux therapies, such as PPIs or P-CAB, and 5-HT1A receptor agonists buspirone which have shown promise in improving LES pressure and esophageal motility (31, 32), can delay or reduce the incidence of cardiopulmonary involvement. Several limitations of our study should be acknowledged. First, the retrospective cross-sectional design precludes causal inference between esophageal dysmotility and pulmonary involvement. Second, the control group consisted of symptomatic patients referred for HRM, which introduces referral bias and may limit the generalizability of between-group comparisons. Third, the sample size was relatively small, and we did not correct for multiple comparisons, increasing the risk of false-positive findings. Fourth, endoscopy and 24-hour pH monitoring were not routinely performed, limiting our ability to correlate manometric findings with objective measures of gastroesophageal reflux. Finally, the single-center design and relatively short disease duration of our cohort may limit the applicability of our findings to broader SSc populations. Recent comprehensive reviews have highlighted the importance of gastrointestinal involvement in SSc and the need for standardized assessment protocols (33, 34).

## Conclusions

In summary, esophageal motility disorders in SSc were significantly associated with older age, symptoms of gastroesophageal reflux, and decreased lung function. In our study, we attempted to explore a more simplified HRM procedure to reduce patient discomfort, such as eliminating the colloid swallow step. Meanwhile, the patients included in this study had relatively short disease durations, yet we have already identified a high prevalence of esophageal motility disorders and observed their correlation with poorer lung function. Therefore, further prospective studies are still needed in the future to clarify whether early esophageal motility disorders are associated with disease progression and prognosis in patients, so as to guide the treatment and comprehensive management of scleroderma.

While our findings provide preliminary evidence for the utility of Chicago Classification v4.0 in characterizing esophageal involvement in Chinese SSc patients, larger prospective studies with healthy controls and longitudinal follow-up are needed to validate these associations and determine their prognostic significance.

## Data Availability

The raw data supporting the conclusions of this article will be made available by the authors, without undue reservation.

## Supplementary material

Supplementary File 1 **Supplementary tables (Tables S1–S3)**: demographics and symptoms, laboratory tests, and manometric data of the study cohort.

Supplementary File 2 **Additional file 1**: Representative high-resolution manometry study in a patient with systemic sclerosis (single water swallow and multiple rapid swallows).

Supplementary File 3 **Additional file 2**: Representative high-resolution manometry study in a patient with systemic sclerosis (rapid drink challenge and upright examination).
