# Supplementary material for: The application of Chicago classification 4.0 in esophageal motility disorders of scleroderma: a case-control study in China
Source: Front Immunol. 2026 Oct 5;17:1962332. doi: 10.3389/fimmu.2026.1962332 (PMC13677876; doi:10.3389/fimmu.2026.1962332)
Supplement: Supplementary file 1 [file Table1.docx]

| Table S1 Symptoms of SSc Patients |  |
| --- | --- |
| Symptom |  |
| Globus Sensation | 14 (23.33) |
| Retrosternal Foreign Body Sensation | 4 (6.67) |
| Retrosternal Pain | 7 (11.67) |
| Acid Regurgitation & Heartburn | 24 (40.00) |
| Dysphagia | 16 (26.67) |
| Epigastric Pain | 13 (21.67) |
| Epigastric Bloating | 13 (21.67) |
| Belching | 12 (20.00) |
| Nausea | 10 (16.67) |
| RDQ(scores) |  |
| Median (IQR) | 2.50 (0.00-7.25) |
| GERDQ(scores) |  |
| Median (IQR) | 1.00 (0.00-4.25) |
| Table S2 Laboratory test of SSc patients | [Median (IQR)] |
| WBC | 6.31 (5.06-8.41) |
| Hb | 124.00 (113.00-135.00) |
| Platelet | 247.00 (215.50-299.50) |
| Albumin | 38.70 (35.25-41.55) |
| Serum Prealbumin | 211.00 (180.75-245.75) |
| Blood Glucose | 4.73 (4.26-5.51) |
| Creatinine | 52.00 (48.00-64.00) |
| Serum Uric Acid | 326.00 (257.00-409.00) |
| Cystatin C | 1.01 (0.86-1.18) |
| β2-Microglobulin | 2.53 (1.90-3.21) |
| Cholesterol | 4.70 (3.84-5.50) |
| Triglycerides | 1.32 (1.05-1.74) |
| High-Density Lipoprotein | 1.01 (0.82-1.23) |
| Low-Density Lipoprotein | 2.80 (2.10-3.24) |
| CK | 79.00 (58.25-130.50) |
| IgG | 12.87 (10.89-16.99) |
| C3 | 1.11 (0.98-1.22) |
| C4 | 0.23 (0.18-0.26) |
| CH50 | 48.00 (39.00-57.75) |
| CRP | 1.90 (0.93-5.85) |
| Erythrocyte Sedimentation Rate | 29.00 (14.00-51.00) |
| **Antibodies** |  |
| ANA Titer |  |
| 0 | 6 (10.00) |
| 80 | 5 (8.33) |
| 320 | 20 (33.33) |
| 640 | 9 (15.00) |
| 1280 | 20 (33.33) |
| Anti-dsDNA | 23 (38.33) |
| Anti-C1q Antibody | 28 (46.67) |
| Anti-Nucleosome Antibody | 4 (6.67) |
| Anti-Histone Antibody | 1 (1.67) |
| Anti-SM | 3 (5.00) |
| Anti-U1 RNP Antibody | 24 (40.00) |
| Anti-SSA | 17 (28.33) |
| Anti-SSB | 2 (3.33) |
